# Small RNAs, Degradome, and Transcriptome Sequencing Provide Insights into Papaya Fruit Ripening Regulated by 1-MCP

**DOI:** 10.3390/foods10071643

**Published:** 2021-07-15

**Authors:** Jiahui Cai, Ziling Wu, Yanwei Hao, Yuanlong Liu, Zunyang Song, Weixin Chen, Xueping Li, Xiaoyang Zhu

**Affiliations:** Guangdong Provincial Key Laboratory of Postharvest Science of Fruits and Vegetables/Engineering Research Center for Postharvest Technology of Horticultural Crops in South China, Ministry of Education, College of Horticulture, South China Agricultural University, Guangzhou 510642, China; jh.cai_chn@outlook.com (J.C.); ziling_ng@163.com (Z.W.); yanweihao@scau.edu.cn (Y.H.); liuyuanlong@scau.edu.cn (Y.L.); songzunyang@163.com (Z.S.); wxchen@scau.edu.cn (W.C.); lxp88@scau.edu.cn (X.L.)

**Keywords:** 1-MCP treatments, microRNA, degradome and transcriptome, papaya ripening, ethylene and auxin

## Abstract

As an inhibitor of ethylene receptors, 1-methylcyclopropene (1-MCP) can delay the ripening of papaya. However, improper 1-MCP treatment will cause a rubbery texture in papaya. Understanding of the underlying mechanism is still lacking. In the present work, a comparative sRNA analysis was conducted after different 1-MCP treatments and identified a total of 213 miRNAs, of which 44 were known miRNAs and 169 were novel miRNAs in papaya. Comprehensive functional enrichment analysis indicated that plant hormone signal pathways play an important role in fruit ripening. Through the comparative analysis of sRNAs and transcriptome sequencing, a total of 11 miRNAs and 12 target genes were associated with the ethylene and auxin signaling pathways. A total of 1741 target genes of miRNAs were identified by degradome sequencing, and nine miRNAs and eight miRNAs were differentially expressed under the ethylene and auxin signaling pathways, respectively. The network regulation diagram of miRNAs and target genes during fruit ripening was drawn. The expression of 11 miRNAs and 12 target genes was verified by RT-qPCR. The target gene verification showed that *cpa-miR390a* and *cpa-miR396* target *CpARF19-like* and *CpERF RAP2-12-like*, respectively, affecting the ethylene and auxin signaling pathways and, therefore, papaya ripening.

## 1. Introduction

Papaya (*Carica papaya* L.) is a popular and widely planted fruit in tropical and subtropical regions due to its unique taste, nutritional benefits, and medicinal benefits [1,2,3]. As one of the classic climacteric fruits, papaya fruits are highly perishable after harvest as a result of rapid ripening and softening and susceptibility to biotic or abiotic stresses [4,5], which usually result in a high percentage of product loss [6,7].

The ethylene antagonist 1-methylcyclopropene (1-MCP) has been widely used to maintain the quality and extend the shelf life of harvested products [8]. By binding to ethylene receptors, 1-MCP can inhibit the release of ethylene, inhibit the respiratory intensity of fruits and vegetables, and delay the ripening of various fruits and vegetables [9,10,11,12]. 1-MCP treatment on papaya at earlier development stages or for long-term/high-concentration treatments will lead to a “rubbery texture” phenomenon, where the fruits are unable to completely soften during later storage, leading to tasteless lack of flavor [13]. RNA-seq analysis has shown that improper 1-MCP treatment severely inhibits cell wall degradation and fruit softening by inhibiting ethylene signal transduction and cell wall metabolism pathways [10]. Integrated analysis of metabolomics and RNA-seq data has shown that various energy metabolites for the tricarboxylic acid cycle, glycolic acid cycle, flavonoids, and phenylpropane pathways were significantly affected by 1-MCP, all of which play important roles in fruit ripening and the softening disorder caused by improper 1-MCP treatment (400 nL·L^−1^·16 h) [14]. However, a deeper understanding of the ripening and softening disorder caused by inappropriate 1-MCP treatment is imperative for further commercial use of 1-MCP.

Ethylene and auxin play critical roles in fruit ripening [15,16,17]. At the transcript level, various transcription factors act as important regulators in the auxin and ethylene signaling pathways and regulate fruit ripening. In persimmon fruit, the *DkERF8/16/18* genes may participate in fruit ripening by accelerating cell wall modification and ethylene biosynthesis [18]. In papaya, it was found that CpNAC3 interacted with CpMADS4 and regulated the role of ethylene signal transcription factors, namely CpERF9 and CpEIL5, to regulate fruit ripening [19]. CpEBF1 interacts with CpMADS1 and regulates cell wall degradation-related genes to modulate the fruit ripening process and softening disorder caused by 1-MCP [13]. Exogenous auxin delays the ripening process of tomato fruits by inhibiting the production of ethylene, the accumulation of carotenoids, and the degradation of chlorophyll [20]. Auxin-induced *DzARF2A* expression was confirmed in response to exogenous auxin application, indicating the auxin-mediated *DzARF2A* role in durian fruit ripening [21]. Through transcriptome sequencing, it was found that most of the differentially expressed genes between suitable and improper 1-MCP treatment groups were enriched in starch and sucrose metabolism, carbon metabolism, plant hormone signal transduction, and amino acid biosynthesis pathways [10]. Among these differentially expressed genes, there were 21 genes enriched in ethylene and auxin signaling pathways [10].

MicroRNA (miRNA) is a type of non-coding small RNA that is around 21 nucleotide (nt). They are widely presented across eukaryotes, where most are negative regulators of gene expression [22,23]. They mainly regulate the expression of plant genes at the post-transcriptional level by mediating cleavage of mRNA target molecules or reducing the translation of target molecules, thereby regulating morphogenesis, growth, development, plant organ hormone secretion, plant organ signal transduction, and the ability to respond to stressors in the environment [24,25,26]. Recently, a growing body of studies have found that small RNAs were involved in regulating auxin and ethylene signal transduction. Li et al. [27] found that *miR160*, *miR390*, and their target genes were related to auxin signaling and participate in the pathway of adventitious root formation in “M9-T337” apple rootstock. Auxin response factor (ARF) is a plant-specific transcription factor that mediates the downstream expression of auxin response genes by binding to auxin response elements and participating in various processes during plant growth and development [28]. The gene families *TaARF1*, *TaARF4*, *TaARF7*, *TaARF34*, and *TaARF39*, located in the wheat genome, were predicted to be targets of Tae-miRNA160, a stress-responsive miRNA. This showed that they were under the regulation of early auxin-responsive gene expression in the auxin signaling pathway [29]. An analysis of the differential expression of miRNAs in banana under ethylene treatment found that *miR162a*, *miR167a*, *miR172a*, and *miR319a* participated in ethylene-dependent fruit ripening [30]. All these results suggested that miRNAs play critical roles in plant development and fruit ripening by regulating ethylene targets, auxin, and other signaling pathways.

This study integrates a differential expression analysis of miRNA, RNA-seq, and degradation sequencing in papaya fruit under different 1-MCP treatments in an effort to identify the key miRNAs and their targets that regulate papaya fruit ripening and the ripening disorder. This work further explores the transcriptional regulation network in papaya fruit and provides new ideas for future physiological and molecular research.

## 2. Materials and Methods

### 2.1. Plant Material and Treatment

Papaya (*Carica papaya* L. cv. ‘suiyou-2’) fruits were harvested from a local commercial farm in the Panyu district of Guangzhou, Guangdong, China. The fruit were harvested at the color-breaking stage of maturity (5% yellow < peel color < 15% yellow) as long as they were disease-free, packaged into polystyrene boxes, and then transported immediately to the laboratory. The harvested fruit were dipped in 0.2% (*w/v*) hypochlorite solution for 10 min and then soaked in a 500 mg·mL^−1^ solution of mixed prochloraz (Huifeng, Jiangsu, China) and iprodione (Kuaida, Jiangsu, China) for 1 min to minimize the effect of microbes/microbe contamination. Treatments were then carried out with the same protocol as in our previous work in Zhu et al. [31]. A total of 600 papaya fruits were selected and divided into three sub-groups, and each group contained 200 papaya fruits. Two groups of the fruit were treated with 400 nL·L^−1^ 1-MCP in a closed foam box for 2 and 16 h, respectively; air treatment for 16 h was used as a control. All fruits were then treated with 1000 μL·L^−1^ ethephon for 1 min. After air-drying for a few minutes, the papaya fruit was packed in an unsealed polyethylene bag (10 cm × 20 cm, 0.02 mm thick) and stored at 22 °C. All the treatments were conducted with three biological replicates.

Three sampling points at days 0, 1, and 6 were selected for measurement with three biological replicates. For each sampling point, three fruits representing the three biological replicates were collected. Samples were taken from the middle of the papaya fruit flesh, frozen immediately using liquid nitrogen, and then stored at −80 °C for further testing. Subsequent usage included RNA extraction, small RNA sequencing, and degradome sequencing.

### 2.2. RNA Extraction and cDNA Library Preparation

For RNA-seq, all RNA extraction and library preparation procedures were conducted as described in Zhu et al. [31].

The NEB Next Ultra-small RNA Library Preparation Kit for Illumina (NEB, #E7530, Ipswich, MA, USA) was used according to the manufacturer’s protocol in order to generate a small RNA-seq library. Library quality was evaluated using an Agilent Bioanalyzer 2100 system. Samples from the control group, short-term 1-MCP treatment group, and long-term 1-MCP treatment group were selected for miRNA analysis after storage for 0, 1, and 6 days. Each sample time contained three biological replicates, and a total of 21 libraries were constructed. After removing the adapter sequences and low-quality sequence reads (including reads containing more than 10% N, reads without 3′ linker sequences, and sequences shorter than 18 nucleotides or longer than 30 nucleotides), the clean reads were then mapped to the papaya reference genome (http://www.plantgdb.org/CpGDB/). Using a BLASTN search against miRbase (V21), known miRNAs were identified from mapped small RNA tags. For the new miRNA candidates, we used miRDeep2 for their identification. The total number of identified miRNAs in each constructed library was then normalized to TPM (number of transcripts per million of the clean tags). The DEGseq R package was used for differential expression analysis between the different groups. The q-value was used to adjust the *p*-value. Small RNAs with |log2(foldchange)| > 1 and *p* -value < 0.01 were assigned as differentially expressed. Target Finder software was used to predict the target genes of differentially expressed miRNAs (http://targetfinder.org/). The target gene identification was also based on the integration of small RNAs and transcriptome sequencing analysis. Normally, miRNA and mRNA pairs have a negative regulatory network relationship regarding their expression. GO software (http://www.geneontology.org/) was used to enrich and analyze the differential target genes between sample groups, and the KEGG (Kyoto Encyclopedia of Genes and Genomes) database was used to analyze the pathway annotation of differentially expressed miRNA target genes (http://www.genome.jp/kegg/).

The papaya samples, including fruits treated with 400 nL·L**^−1^** 1-MCP for 2 or 16 h and from the control group, were mixed and used to establish a degradation group library. After the samples were extracted, total RNA was extracted using the reagent Trizol (Invitrogen, CA, USA). The quantity and purity of the total RNA were analyzed using a Bioanalyzer 2100 and RNA 6000 Nano Lab Chip Kit (Agilent, CA, USA) with an RIN number > 7.0. Approximately 20 µg of total RNA was used for degradome library construction. Single-end sequencing (36 bp) was performed on an Illumina Hiseq2500 at LC-BIO (Hangzhou, China) according to the protocol recommended by the sequencing facility. Raw sequencing reads containing no adaptors nor low-quality reads were obtained using Illumina’s software. The filtered sequencing reads were then used to identify potentially cleaved targets using the CleaveL pipeline. Degradome data and reads were mapped to the mRNA downloaded from JGI (http://www.plantgdb.org/CpGDB/). Only perfectly matched alignments to the given read were preserved for further degradation analysis.

### 2.3. RT-qPCR Verification

In order to verify the expression profiles of the identified miRNA and its target mRNA, 7 known miRNAs, 4 novel miRNAs, and 12 corresponding target genes were selected for real-time quantitative PCR (RT-qPCR) validation.

Total RNA and small RNAs were extracted using TRIzol reagent and Fruit-mate for RNA purification (Takara, Japan). Total RNA was reverse-transcribed using stem-loop qRT-PCR [32]. Stem-loop primers for reverse transcription and primers for RT-qPCR are listed in Tables S1 and S2. The relative gene expression value was normalized against the relative value of the *CpTBP1* gene for mRNA expression [33] and 5 s RNA for miRNA expression [34]. SuperScript III reverse transcriptase (Invitrogen, Carlsbad, California, USA) was used to reverse transcribe the total RNA using the pulse reverse transcription program. The RT-qPCRs were performed using a total volume of 20 µL containing 10 µL SYBR Mixture (Promega, Madison, Wisconsin, USA), 3 µL cDNA template, 0.5 mM primers, and 6 µL ddH2O. PCR was performed on a Bio-Rad CFX96 real-time PCR system using the qPCR Master Mix Kit (Promega, Madison, WI, USA). Three biological replicates were used to determine the expression of each gene, and expression was calculated using the 2^−ΔΔCT^ method [33].

### 2.4. Plant Expression Vector Construction and Transformation

Validated miRNAs were cloned into a pBI121 binary vector driven by a cauliflower mosaic virus 35S promoter (CaMV35S). The miRNAs’ corresponding target genes were cloned into a pMS4 vector containing green fluorescent protein (GFP). Vector constructions were conducted in reference to Wang’s method [35]. Confirmation of the target gene site was identified using the website http://plantgrn.noble.org/psRNATarget/). The primers are listed in Table S3. The constructed vectors were then transformed into tobacco mediated by *Agrobacterium tumefaciens* (GV3101). Young tobacco leaves were cultivated for five weeks and used for injection of *Agrobacterium tumefaciens*. Transformed leaves were photographed with ultraviolet light after 36 h of infection.

### 2.5. Statistical Analysis

Each treatment was conducted in three independent biological replicates, and the variance of the collected data was analyzed. Statistical differences between groups were assessed based on Duncan’s Multiple Range Test (DMRT) in SPSS 19.0 (IBM, Armonk, NY, USA). The graphics were drawn using Prism 8 software (GraphPad Inc., La Jolla, CA, USA) and Adobe Illustrator software (CC 2017; Adobe Inc., San Jose, CA, USA).

## 3. Results

### 3.1. Construction and Sequencing of Small RNA Libraries

**Figure 1:**
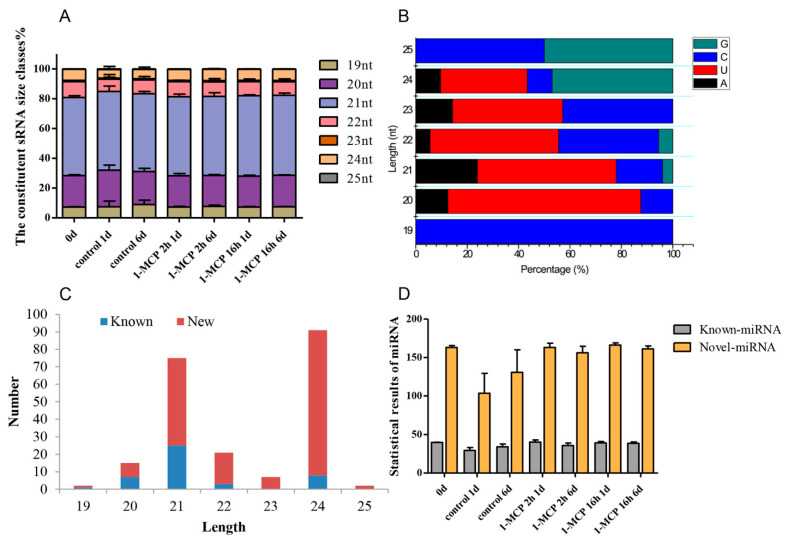
Changes in the distribution and complexity of sRNA in papaya samples. (**A**) Sizes of the 19–25 nt sRNA readings are classified and distributed under different 1-MCP treatments. The composition of sRNAs at each storage time point is shown, defined as 0 d, 1-MCP 2 h 1 d and 6 d, and 1-MCP 16 h 1 d and 6 d. The rRNA and tRNA sequences were deleted. (**B**) Base preference distribution of different sRNA reads. (**C**) sRNA read length classification distribution of known and novel miRNAs. (**D**) Statistical results of miRNA for each sample, including known and novel miRNAs.

Our previous work showed that 1-MCP treatment (400 nL·L^−1^, 2 h) can delay the ripening of papaya fruit, but improper 1-MCP treatment (400 nL·L^−1^, 16 h) causes an adverse “rubbery” texture [31]. Therefore, small RNA (sRNA) sequencing was performed. A total of 354.06 megabytes (MB) of clean reads was obtained from 21 different libraries. For each sample, low-quality sequences and reads with an unknown base (N) greater than 10% of the sequence were first removed and then 3′ adaptor sequences, reads with low complexity, and reads homologous to t/rRNA sequence were removed. The data for each sample consisted of no less than 12.23 MB of clean reads (Table S4). In addition, 98.77 to 99.11% of the total reads ranging from 18 to 30 nt library lengths exactly matched the papaya genome sequence. The length distribution of the sRNAs was mainly concentrated between 18 and 24 nt, where the papaya libraries were mainly composed of 20 and 21 nt length sRNAs (Figure 1A). However, in the papaya injected with papaya ringspot virus (PRSV), 21 and 24 nt length sRNAs accounted for the majority of the total sRNAs present [36]. One possible reason for the observed difference in mature sRNA lengths across the different groups may be due to the differential activity across the various sRNA biogenesis pathways. Nucleotide sequence analysis of the identified miRNAs showed that uridine (U) was the most common nucleotide in sRNAs of 20–24 nt length, while cytosine (C) had a higher enrichment in the sRNAs of 19 and 25 nt lengths (Figure 1B). C and U account for the majority of the total number of bases in papaya sRNA.

### 3.2. Identification of Known and Novel miRNAs

Known miRNAs in papaya fruit were identified through sequence matching with an miRNA database (miRBase v21). Read sequences that were exactly the same as the known miRNA were considered to be an identified known miRNA. A total of 213 miRNAs were identified in the present study, 44 of which are already known miRNAs, and 169 are newly predicted miRNAs (Table S5). As shown in Figure 1C, the distribution of known and new miRNAs in sRNAs is different across different lengths. The number of new miRNAs discovered was significantly greater than the number of known miRNAs. Among the newly discovered miRNAs, the number of 21 and 24 nt length miRNAs was significantly greater than that of those of other size classes. The distribution of known miRNAs and new miRNAs from each sample is shown in Figure 1D. Fewer miRNAs were identified in the 1 day (s) after treatment (DAT) and 6 DAT samples in the control than in the other samples. Among all the known miRNAs, 14 miRNAs showed high abundance > 5000 TPM, including *cpa-miR159a*, *cpa-miR159b*, *cpa-miR160d*, *cpa-miR162a*, *cpa-miR164a*, *cpa-miR166a*, *cpa-miR166d*, *cpa-miR319*, *cpa-miR390a*, *cpa-miR396*, *cpa-miR477*, *cpa-miR8137*, *cpa-miR8141*, and *cpa-miR8148*. Five identified miRNAs from across all the miRNA libraries showed low abundance < 100 TPM, including *cpa-miR160c-3p*, *cpa-miR164d*, *cpa-miR5211*, *cpa-miR8134*, and *cpa-miR8150*. In addition, 169 novel miRNA candidates from 21 different libraries were predicted. It seems that 25.4% of the novel miRNA candidates showed lower abundance < 500 TPM. Among all the identified novel miRNAs, *unconservative_supercontig_106_26263* and *unconservative_supercontig_17_8265* were in greatest abundance (Table S6).

### 3.3. Identification of Differentially Expressed miRNAs

**Figure 2:**
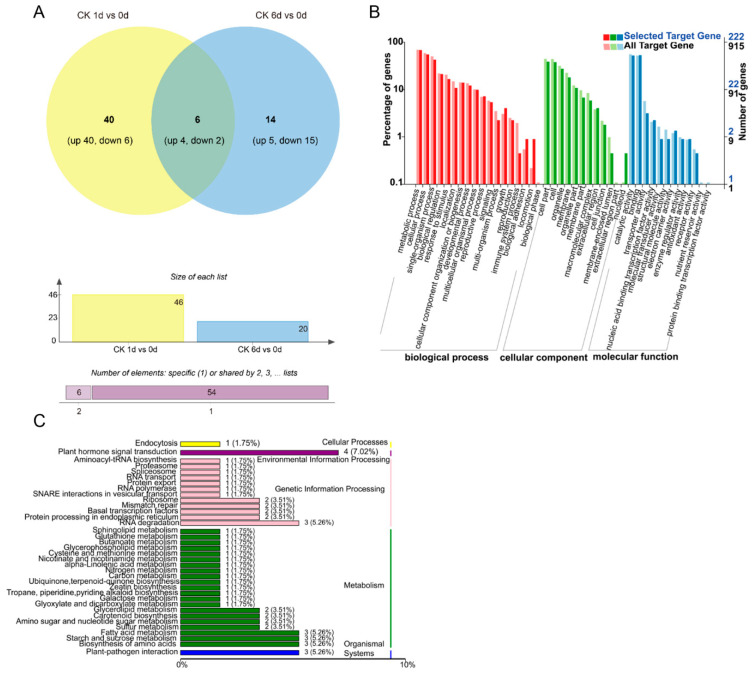
DEM (differentially expressed miRNA) analysis under control conditions during the fruit ripening stage. (**A**) Number of DEMs in papaya fruit at 1 and 6 days after treatment (DAT) compared to 0 DAT under control conditions. The Venn software package (http://bioinformatics.psb.ugent.be/webtools/Venn/) was used for the Venn diagram. (**B**) GO classification of the predicted target genes in the comparison of 1 and 6 DAT with 0 DAT. (**C**) KEGG classification of the predicted targets of target genes in the comparison of 1 and 6 DAT with 0 DAT.

Compared to 0 DAT, 60 differentially expressed miRNAs (DEMs) (|log2(FC)| ≥ 1, *p*-value ≤ 0.05) were identified (at 1 DAT vs. 0 DAT and 6 DAT vs. 0 DAT) (Figure 2A). There were 46 DEMs identified between 1 and 0 DAT in the control group, of which 40 DEMs showed increased expression and 6 DEMs showed decreased expression (Figure 2A). There were 20 DEMs identified between 6 and 0 DAT in the control group, among which 5 DEMs had increased expression and 15 DEMs had decreased expression. Among the DEMs in both comparison groups, there were six DEMs that were shared across both comparisons during the papaya ripening stage, of which four DEMs had increased expression and two DEMs had decreased expression during fruit ripening. The number of DEMs decreased during fruit ripening (Figure 2A). The enriched GO terms for the predicted target genes are presented in Figure 2B. “Biological process of metabolic process”, “single-organism process and cellular process”, “the cellular component of cell part and cell”, and “the molecular function of catalytic activity and binding” had the most significant differences in gene expression changes during papaya fruit ripening. In the KEGG enrichment analysis, there were 36 top enrichments for metabolic/biological pathways (Figure 2C). Among these enriched genes, “metabolic pathways of plant hormone signal transduction”, “fatty acid metabolism”, “biosynthesis of amino acids”, and “starch and sucrose metabolism” are important for fruit ripening.

**Figure 3:**
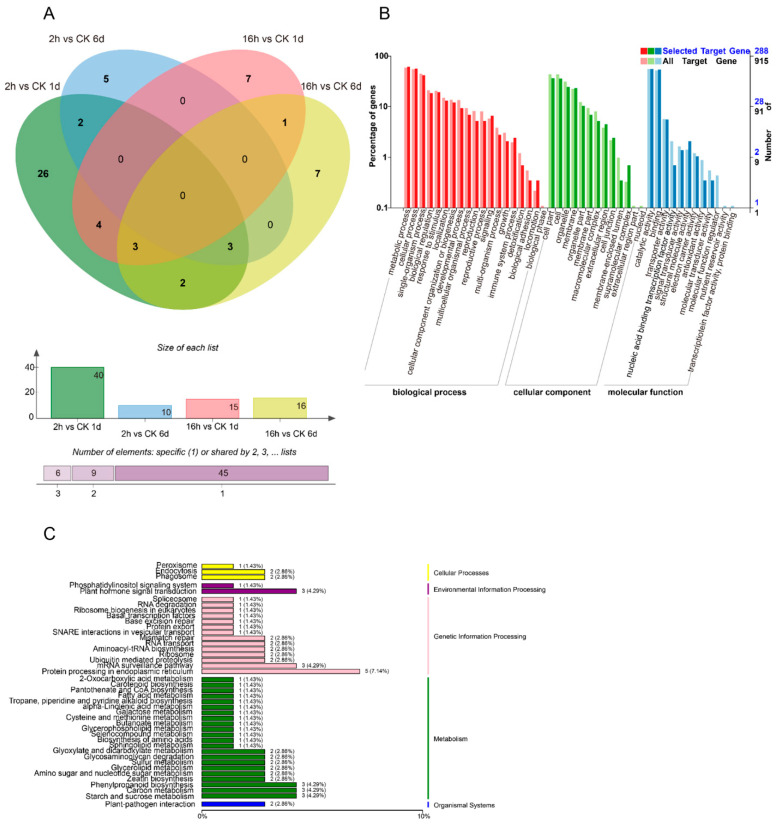
The effect of different 1-MCP treatments on the expression of miRNA. (**A**) The number of DEMs (differential expressed miRNAs) derived from comparison between the 1-MCP treatments (2 h) in 1- and 6-day samples and the control samples at each time point, and the number of DEMs derived from comparison between the 1-MCP treatments (16 h) in 1- and 6-day samples and the control samples at each time point. The Venn software package (http://bioinformatics.psb.ugent.be/webtools/Venn/) was used for the Venn diagram. (**B**) GO classification of the predicted targets of target genes in the 1-MCP (16 h) 1- and 6-day samples compared to the control, and the 1-MCP (2 h) 1- and 6-day samples compared to the control at each time point. (**C**) KEGG classification of the predicted targets of target genes in the 1-MCP (16 h) 1- and 6-day samples compared to the control, and the 1-MCP (2 h) 1- and 6-day samples compared to the control at each time point.

Figure 3A shows a total of 60 miRNAs that were differentially expressed between 1-MCP treatments (long-term and short-term) compared to the control (Figure 3A). The amount of DEMs decreased with the storage time and duration of the 1-MCP treatment. Compared to the control, a total of 45 miRNAs were differentially expressed during short-term 1-MCP treatment. There were 35 DEMs specifically identified in the comparison of 1-MCP 2 h vs. CK at 1 DAT, 5 DEMs specifically identified in the comparison of 1-MCP 2 h vs. CK at 6 DAT, and 5 common DEMs shared across both DATs. Contrary to this, a total of 27 DEMs were identified in the comparison of long-term 1-MCP vs. the control. Of these, 11 and 12 DEMs were specifically identified after 1 and 6 DAT, respectively, and 4 DEMs were shared across both DATs. These results indicate that miRNAs are important in regulating fruit ripening.

In the abnormal fruit ripening case caused by 1-MCP treatment, the number of DEMs decreased dramatically compared to short-term 1-MCP treatment, indicating that the missing DEMs may be involved in the softening disorder. In order to fully understand the functions of DEM target genes, a GO enrichment analysis was performed (Figure 3B). The enrichments for “metabolic process and cellular process”, “the cellular component of cell part and cell”, and “molecular function of catalytic activity and binding” biological processes showed the most significant differences in gene expression during 1-MCP-treated papaya fruit ripening. From the KEGG enrichment terms, there were 38 top enrichments for metabolic/biological pathways (Figure 3C). Among these KEGG enrichment terms, “metabolic pathways of plant hormone signal transduction”, “phenylpropanoid biosynthesis”, “protein processing in endoplasmic reticulum”, “carbon metabolism”, and “starch and sucrose metabolism” genes were the most enriched, indicating the important roles of these terms in fruit ripening and 1-MCP-caused ripening disorder. The COG function for different treatments is shown in Figure S1. All DEMs were clustered into 26 functional categories based on four kinds of differential enrichment analyses. Among the 26 functional categories, “general function prediction only”, “transcription”, and “replication, recombination, and repair” were the most enriched items. The signal transduction mechanism was also enriched, which is an important functional protein for the ripening process of papaya (Figure S1).

Ethylene and auxin signaling pathways showed an important role in the ripening of papaya treated with different durations of 1-MCP treatment [31]. According to the perfect or near-perfect complementarity of expression between miRNAs and their targets, DEMs were predicted to correspond to uni-genes as potential targets. Through sRNA sequencing analysis, Target Finder software was used for predicting targets. According to sRNA sequencing, miRNAs and their predicted target genes related to ethylene and auxin were found, and eight miRNAs and their predicted target genes with opposite expression levels were screened out. It indicates that miRNAs may play a critical regulatory role in ethylene and auxin signal transduction pathways. The identified miRNAs and their corresponding target genes are shown in Table 1. In the early development stage of papaya ripening (1 DAT vs. 0 DAT in the control), *cpa-miR319* was downregulated, while *cpa-miR396* and *cpa-miR8140* were upregulated. At the late development stage of papaya ripening (6 DAT vs. 0 DAT in the control), the expression of *cpa-miR160d* decreased. At the late development stage of delayed papaya ripening, *cpa-miR390a* (1-MCP 2 h 6 DAT vs. control 6 DAT) and *cpa-miR396* were upregulated; *cpa-miR172a*, on the other hand, was downregulated (1-MCP 2 h 6 DAT vs. 1 DAT). At the early development stage of papaya softening disorder caused by 1-MCP (1-MCP 16 h 1 DAT to control 1 DAT), *cpa-miR172a* was upregulated. Meanwhile, during the ripening stage of papaya with ripening disorder caused by 1-MCP, the expression of *cpa-miR160d*, *unconservative_supercontig_2_1866* (1-MCP 16 h 6 DAT vs. control 6 DAT), *cpa-miR167c*, and *cpa-miR8140* (1-MCP 16 h 6 DAT vs. 1 DAT) was upregulated. These results indicated that miRNAs may be involved in ethylene and auxin signaling and participate in the ripening of papaya.

### 3.4. Combined Expression Analysis of miRNAs and Their Target mRNAs

**Figure 4:**
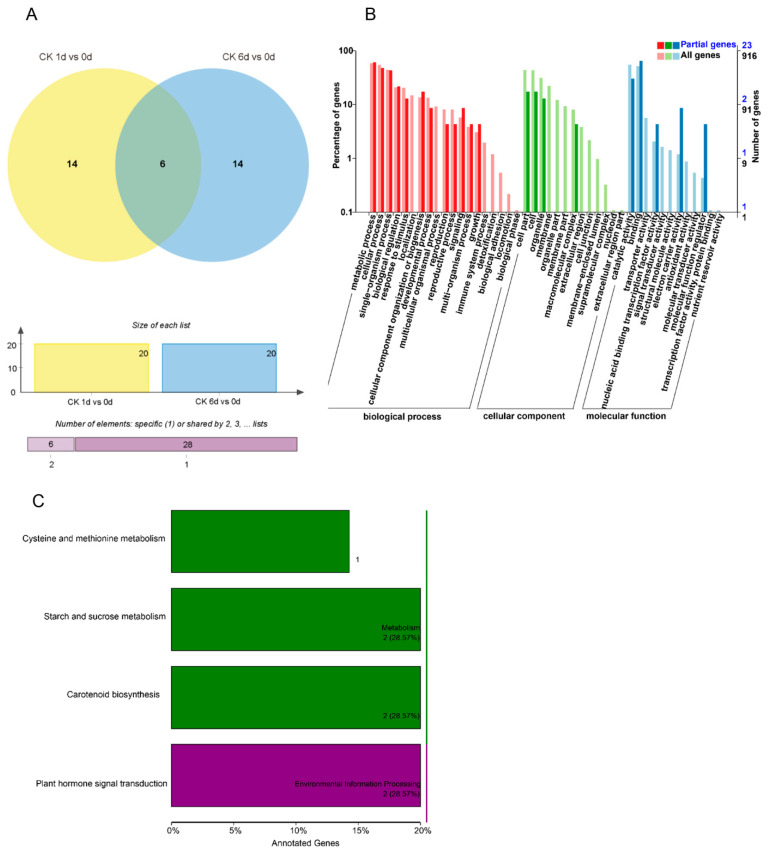
Changes in DEMs (differentially expressed miRNAs) during papaya fruit ripening from both miRNA sequencing and transcriptomic analysis. (**A**) The number of DEMs in papaya fruit at 1 and 6 days after treatment (DAT) compared to 0 DAT under control conditions. (**B**) GO classification of target gene comparison at 1 and 6 DAT compared to 0 DAT. (**C**) KEGG classification of target gene comparison at 1 and 6 DAT compared to 0 DAT.

Through differential expression analysis of the miRNAs and mRNAs in the sRNAs and transcriptome sequencing data, key miRNAs and genes were identified. The regulation of miRNAs and their target genes was uncovered. Compared to 0 DAT, 34 DEMs were identified, of which 20 DEMs were found at both 1 and 6 DAT. Six DEMs were shared across both DAT groups (Figure 4A). The enriched target gene GO terms are presented in Figure 4B. The enrichments for “biological process of metabolic process and cellular process” and “the molecular function of binding” biological processes showed the most significant differences in gene expression during papaya fruit ripening. From the KEGG enriched terms, “metabolic pathways of plant hormone signal transduction”, “carbon metabolism”, and “starch and sucrose metabolism” were important for fruit ripening (Figure 4C).

**Figure 5:**
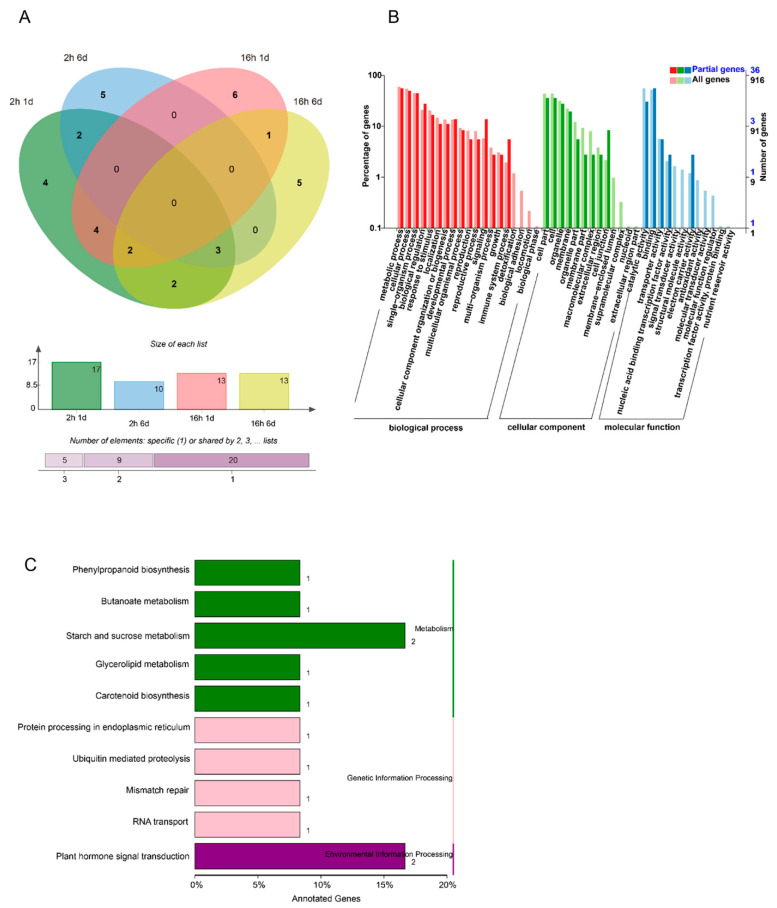
The effect of 1-MCP treatments on both miRNA sequence and transcriptomic analysis. (**A**) The number of DEMs (differentially expressed miRNAs) derived from comparison between the 1-MCP (2 h) treatment and the control on 1- and 6-day samples, and the number of DEMs derived from comparison between the 1-MCP (16 h) treatment and the control on 1- and 6-day samples. (**B**) GO classification of target genes between the 1-MCP (16 h) 1- and 6-day samples and the 1-MCP (2 h) samples at each time point. (**C**) KEGG classification of target genes between the 1-MCP (16 h) 1- and 6-day samples and the 1-MCP (2 h) samples at each time point.

Combined with transcriptome analysis, a total of 34 miRNAs were expressed in the different 1-MCP treatments (long-term and short-term). The amount of DEMs increased with treatment storage time and duration of the 1-MCP treatment. In total, 22 DEMs were identified in the comparison of short-term 1-MCP vs. the control at 6 and 1 DAT. Among the 22 DEMs, 17 DEMs were identified at 1 DAT, 10 were at identified 6 DAT, and 5 were shared across both dates. The enriched GO terms for miRNA target genes are presented in Figure 5B. The enrichments for “metabolic process and cellular process” and “molecular function of binding” biological processes showed the most significant differences in gene expression during 1-MCP-delayed papaya ripening. From the KEGG enriched terms, “metabolic pathways of plant hormone signal transduction” and “starch and sucrose metabolism” were important for fruit ripening (Figure 5C).

Combined with transcriptome analysis, the miRNA target genes were screened out if they had an opposite expression pattern to their respective miRNAs. In total, 12 miRNAs were found to be involved in the signal transduction process of ethylene and auxin. The miRNAs and their corresponding target genes are shown in Table 2. At the early development stage of papaya ripening (1 DAT vs. 0 DAT in the control group), the expression of *cpa-miR319* was downregulated while *cpa-miR396*, *cpa-miR8140*, *unconservative_supercontig_120_28048*, and *unconservative_supercontig_46_16464* were upregulated. At the late development stage of papaya ripening (6 DAT vs. 0 DAT in the control), the expression of *cpa-miR160d* and *unconservative_supercontig_2_1866* was downregulated while that of *unconservative_supercontig_46_16464* was upregulated. At the late ripening stage of papaya fruit under suitable 1-MCP treatment (1-MCP 2 h 6 DAT vs. control 6 DAT), the expression of *cpa-miR390a* was upregulated. During the ripening stage of papaya fruit under suitable 1-MCP treatment, the expression of *cpa-miR172a* (1-MCP 2 h 6 DAT vs. 1 DAT) was upregulated, while the expression of *cpa-miR396* and *unconservative_supercontig_9_5033* was downregulated (1-MCP 2 h 6 DAT to 1 DAT). At the early development stage in papaya with the ripening disorder caused by 1-MCP (1-MCP 16 h 1 DAT vs. control 1 DAT), the expression of *cpa-miR172a* and *unconservative_supercontig_52_17983* was upregulated, and that of *unconservative_supercontig_120_28048* was downregulated. At the late development stage in papaya with ripening disorder, *cpa-miR160d* and *unconservative_supercontig_2_1866* were upregulated (1-MCP 16 h 6 DAT to control 6 DAT). During the papaya ripening stage for individuals with the ripening disorder, the expression of *cpa-miR167c* and *cpa-miR8140* was upregulated (1-MCP 16 h 6 DAT to 1 DAT).

### 3.5. Target Gene Identification of Papaya miRNAs by Degradome Analysis

**Figure 6:**
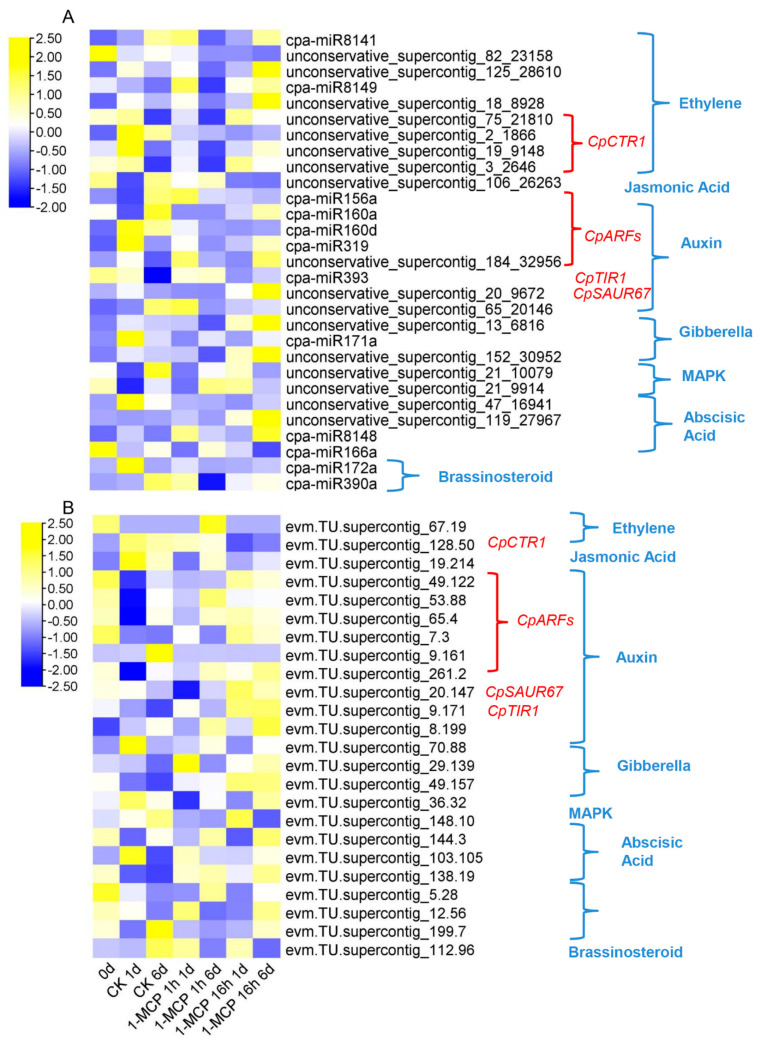
Expression profiles of DEMs (differentially expressed miRNAs) and their candidate target genes in hormone signal pathways. (**A**) Heat map display of DEM expression profiles involved in plant hormone signaling metabolic pathways under 1-MCP treatment combined with the degradation analysis. (**B**) Heat map display of the candidate target genes of DEMs involved in plant hormone signaling metabolic pathways under 1-MCP treatment combined with the degradation analysis.

Through degradome sequencing, the splice sites of miRNAs in the mRNA were found. The 1741 target genes corresponding to their miRNAs are summarized in Table S7. According to KEGG classification, the target genes of these key DEMs related to plant hormone signal transduction are summarized and shown in Figure 6A,B. The plant hormone signal pathways include ethylene, jasmonic acid, auxin, gibberella, MAPK, and abscisic acid. Among all these pathways, the miRNAs related specifically to the ethylene and auxin signaling pathways were the most enriched. The corresponding key target genes were *CpCTR1*, *CpARFs*, *CpTIR1*, and *CpSAUR67*. The expression of some miRNAs increased with fruit ripening, such as *unconservative_supercontig_106_26263*, *cpa-miR156a*, and *cpa-miR160d* and *cpa-miR160a*. Some miRNAs were closely related to papaya softening disorder, such as *unconservative_supercontig_20_9672*, *unconservative_supercontig_13_6816*, *cpa-miR8148*, *unconservative_supercontig_152_30952*, and *unconservative_supercontig_119_27967*. Four miRNA targets to *CpCTR1* were identified: *unconservative_supercontig_75_21810*, *unconservative_supercontig_2_1866*, *unconservative_supercontig_19_9148*, and *unconservative_supercontig_3_2646*. *CpSAUR67* and *CpTIR1* were involved in auxin biosynthesis and signal transduction and were putative targets for *unconservative_supercontig_20_9672* and *cpa-miR393*, respectively. *CpARFs* were putative targets for *cpa-miR160d*, *cpa-miR319*, *unconservative_supercontig_184_32956*, *cpa-miR156a*, and *cpa-miR160a*. The expression of these target genes was neither increased with fruit ripening and repressed by 1-MCP treatment nor decreased with fruit ripening, but it was induced by 1-MCP treatments (Figure 6B), indicating that these target genes may play an important role in the fruit ripening process.

### 3.6. Target Gene Identification of Papaya miRNAs by Degradome Analysis

**Figure 7:**
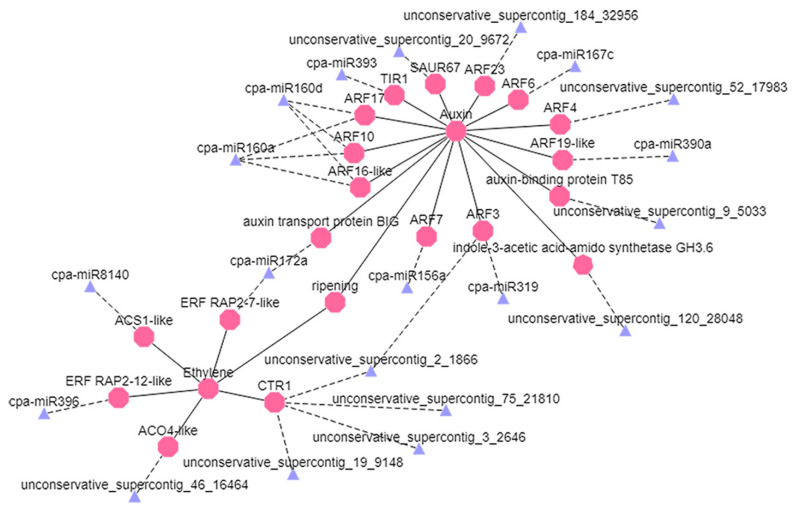
Regulation network diagram of miRNAs and their candidate target genes. Blue triangles represent the miRNAs, pink polygons represent the corresponding target genes, the dotted line shows negative regulation, and the straight line shows positive regulation.

According to the analysis of small RNAs, degradome, and transcriptome sequencing, the miRNAs related to ethylene and auxin signaling were selected and visualized. The *cpa-miR160a* and *cpa-miR160d* miRNAs co-targeted *CpARF10*, *CpARF16-like*, and *CpARF17*, thereby affecting auxin signaling and fruit ripening. The *cpa-miR172a* miRNA targeted *CpERF RAP2-7* and *Carica papaya auxin transport protein BIG*, and *unconservative_supercontig_2_1866* targeted *CpARF3* and *CpCTR1*, thereby affecting the signaling pathways of ethylene and auxin simultaneously (Figure 7).

### 3.7. Target Gene Identification of Papaya miRNAs by Degradome Analysis

**Figure 8:**
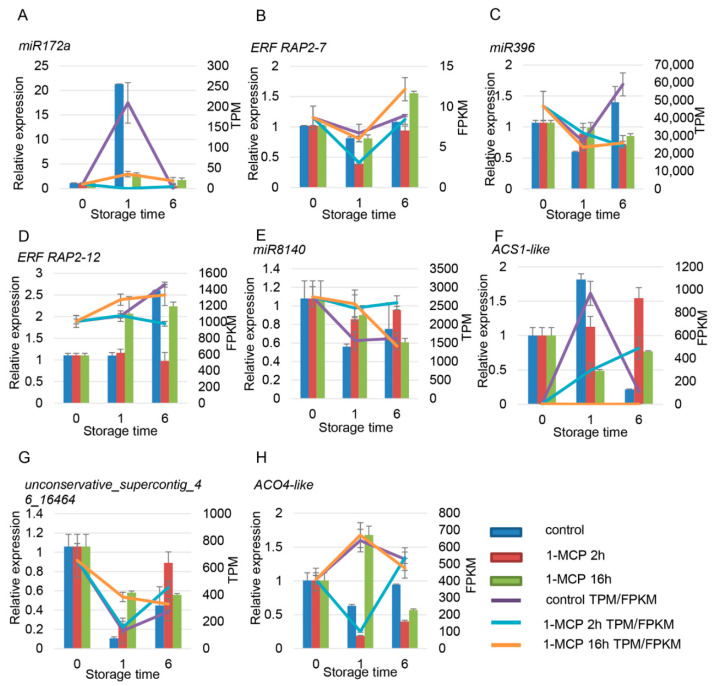
Expression pattern validation of selected DEMs (differentially expressed miRNAs) and their target genes related to the ethylene signaling pathway under different 1-MCP treatments. The histograms were plotted using data obtained from RT-qPCR, and the corresponding line chart was plotted using TPM/FPKM values from the RNA-seq analysis. (**A**–**H**) are miRNA and the their target gene pairs. (**A**,**C**,**E**,**G**) are the miRNA, and (**B**,**D**,**F**,**G**) are the corresponding target gene, respectively. Different colors indicate different samples. The expression of samples at 0 DAT was set to 1. *ACT* and *TBP1* were used as references, which were validated by Zhu et al. (2012).

Through the analysis of sRNAs and mRNA sequencing, the miRNAs and their target genes that are related to ethylene and auxin were selected and verified by RT-qPCR. There were four miRNAs related to ethylene signaling, namely *cpa-miR172a*, *cpa-miR396*, *cpa-miR8140*, and *unconservative_supercontig_46_16464*. Among these miRNAs, the expression of *miR172a* in the control group increased sharply at 1 DAT and then decreased. 1-MCP treatments severely repressed the expression of *miR172a* (Figure 8A). *CpERF RAP2-7-like* was predicted to be the target gene of *miR172a*. The expression of *CpERF RAP2-7-like* decreased at 1 DAT and then increased at 6 DAT, with an opposite expression trend compared to *miR172a* (Figure 8B). The expression of *cpa-miR396*, *cpa-miR8140*, and *unconservative_supercontig_46_16464* decreased with fruit ripening, while the expression of their corresponding target genes, namely *CpERF RAP2-12-like*, *CpACS1-like*, and *CpACO4-like*, increased first and then decreased, respectively (Figure 8C–H). Among the four ethylene-related genes, the expression level of *CpACS1* significantly increased at 1 DAT in the control sample, signaling that these genes may play an important role in the early development stage of fruit ripening. The expression levels of *CpACO4*, *CpERF4*, and *CpERF RAP2-7-like* slightly decreased and were negatively correlated with fruit ripening. The expression of *CpERF RAP2-12-like* increased with fruit ripening and was positively correlated with fruit ripening (Figure 8). The RT-qPCR results were consistent with the RNA-seq data, and miRNA and the corresponding target genes showed opposite expression patterns.

### 3.8. Target Gene Identification of Papaya miRNAs by Degradome Analysis

**Figure 9:**
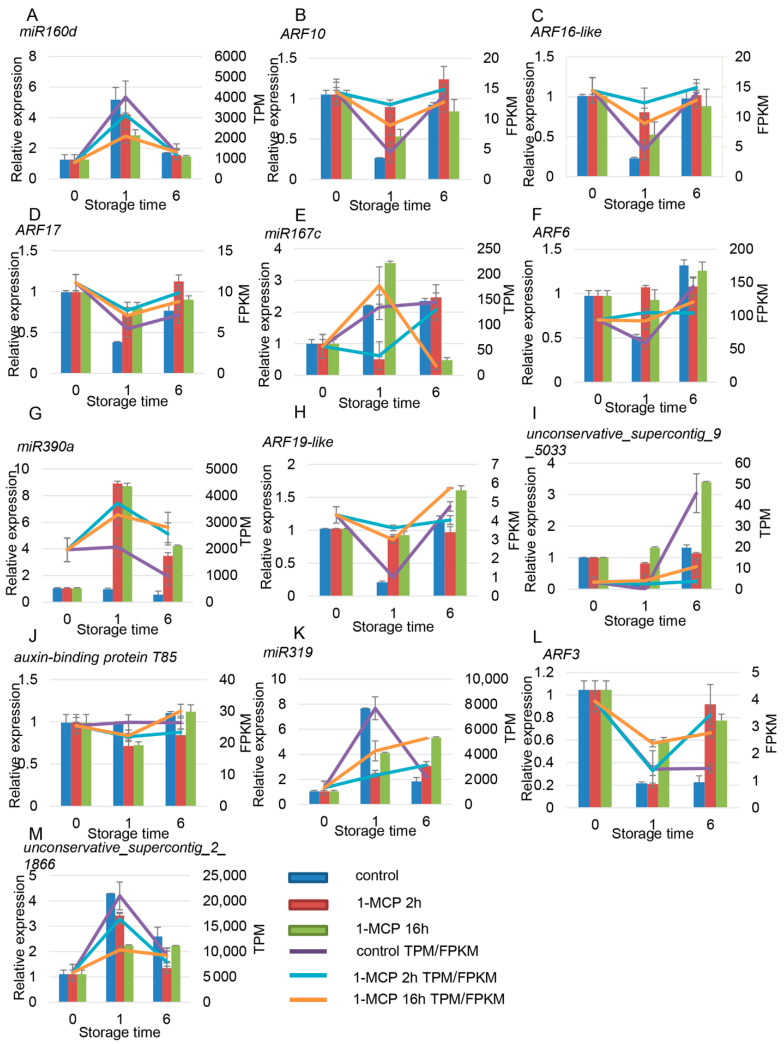
Expression pattern validation of selected DEMs (differentially expressed miRNAs) and their target genes related to the auxin signaling pathway under different 1-MCP treatments. The histograms were plotted using data obtained from RT-qPCR, and the corresponding line chart was plotted using TPM/FPKM values from the RNA-seq analysis. (**B**–**D**) are the target genes of (**A**); (**F**) is the target genes of (**E**); (**H**) is the target genes of (**G**); (**J**) is the target genes of (**I**); (**L**) is the target genes of (**K**,**M**). Different colors indicate different samples. The expression of samples at 0 DAT was set to 1. *ACT* and *TBP1* were used as references, which were validated by Zhu et al. (2012).

There were six miRNAs and seven target genes identified related to auxin signaling. Among the miRNAs and target genes, miR160a and miR160d worked together to regulate the expression of *ARF10/16-like/17*. Similar expression profiles in *miR160d*, *miR167c*, *miR319*, *miR390a*, and *unconservative_supercontig_2_1866* were observed, which first increased and then decreased (Figure 9A,E,G,K). On the contrary, expression of the target gene ARF first decreased and then increased (Figure 9B–D,F,H,L). The expression of *unconservative_supercontig_9_5033* first decreased and then increased; the expression of its target gene, *auxin-binding protein T85*, increased first and then decreased (Figure 9I,J). The expression of all six of these miRNAs was positively correlated with fruit ripening, but their targets showed a negative correlation with fruit ripening. 1-MCP treatment repressed the expression of these miRNAs but induced the expression of their target genes (Figure 9).

### 3.9. Target Gene Identification of Papaya miRNAs by Degradome Analysis

**Figure 10:**
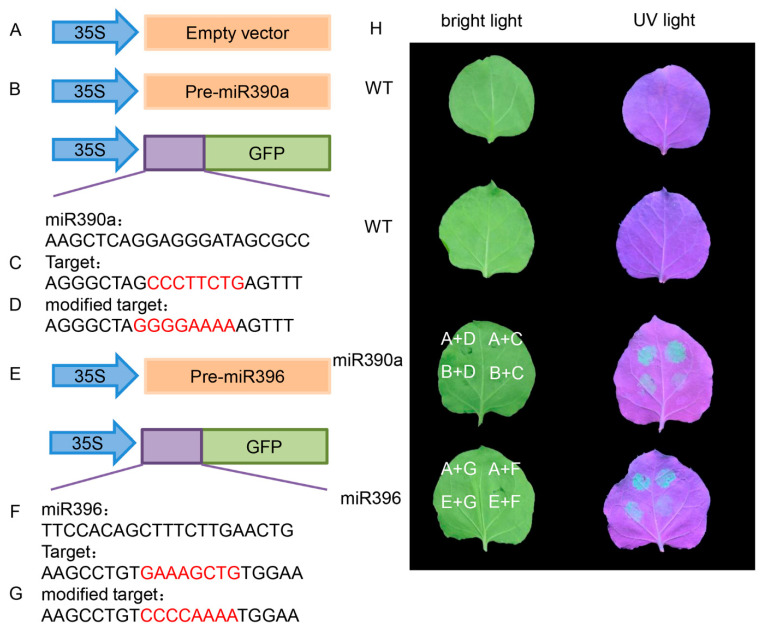
Confirmation of miRNAs and their targets in tobacco. (**A**–**G**) Diagrams of the plasmids used in these experiments. (**A**) Empty vector; (**B**) 35s::pre-miR390a vector that overexpresses Cpa-miR390a; (**C**) 35s::CpARF 19-like-GFP vector that overexpresses CpARF19-like; (**D**) 35s::MCpARF 19-like-GFP vector that overexpresses GFP carrying a modified target; (**E**) 35s::pre-miR396 vector that overexpresses Cpa-miR396; (**F**) 35s::CpERF RAP2-12-like-GFP vector that overexpresses CpERF RAP2-12-like; (**G**) 35s::MCpARF RAP2-12-like-GFP vector that overexpresses GFP carrying a modified target; (**H**) GFP picture of tobacco.

According to the previous analysis, *CpARF19-like* and *CpERF RAP2-12-like* were potential targets of cpa-miR390a and cpa-miR396, respectively. To further test the relationship between these miRNAs and their target genes, transient co-expression assays in *Nicotiana benthamiana* leaves were conducted. As shown in Figure 10, the green fluorescent protein (GFP) fluorescence from tobacco leaf areas injected with 35s::Pre-cpa-miR390a + 35s::CpARF19-like-GFP and 35s::Pre-cpa-miR396 + 35s::CpERF RAP12-2-like-GFP was starkly lower than that of the negative controls (Figure 10). These results provide evidence that the *CpARF19-like* and *CpERF RAP2-12-like* genes may be directly regulated by cpa-miR390 and cpa-miR396 miRNA, respectively.

## 4. Discussion

### 4.1. Papaya miRNAs with Conserved and New Gene Targets

As an inhibitor of ethylene receptors, 1-MCP can effectively delay fruit ripening. This has wide applicability to post-harvest fruit preservation. Improper 1-MCP treatment can negatively affect the softening ability of fruit during ripening, resulting in papaya ripening disorder [37,38,39]. 1-MCP function can reduce the production of endogenous ethylene. In banana, 12 novel and 128 known miRNAs were identified. Among these miRNAs, 22 were differentially expressed after ethylene regulation treatment and were involved in the ethylene response [30]. In Wang’s (2020) study between wild-type and *LeERF1* transgenic tomato fruits, 9 miRNAs and 12 nat-siRNAs were found to be differentially expressed [40]. Degradome sequencing analysis further validated the nine miRNA targets, and six new targets were also identified. In our present study, 46 known miRNA families and 169 papaya-specific miRNAs were identified in 1-MCP-treated papaya samples using deep sequencing and computational analyses (Figure 1, Table S5). This difference in these two studies may be due to the type of fruit and the difference in post-harvest treatments. In papaya, a total of 1741 target genes of 178 known miRNAs were identified through degradome analysis (Table S7). Across the identified miRNAs, there were 40 known miRNAs, 138 novel miRNAs, and 29 miRNAs related to plant hormone signaling. 

### 4.2. miRNAs Participate in Hormone Pathways during Papaya Fruit Ripening

Using miRNA sequencing analysis, 34 miRNAs related to papaya ripening and 60 miRNAs related to 1-MCP treatment were found (Figure 2 and Figure 3). After target prediction (Table 1) and combined analysis of miRNAs and miRNA sequences, 12 miRNAs were found to directly act on ethylene- and auxin-related pathways. Of these 12 miRNAs, eight were known miRNAs and one was a novel miRNA. miRNAs are negative regulators of expression of their target genes. The miRNA mdm-miR160 targets the auxin response factors *MdARF16* and *MdARF17* and participates in the formation of adventitious root in apple rootstocks [27]. During papaya’s ripening stage (6 DAT vs. 0 DAT in the control), the expression of *cpa-miR160d* decreased, while in papaya fruit with abnormal ripening (1-MCP 16 h 6 DAT vs. control 6 DAT), the expression of *cpa-miR160d* increased. These results indicate that *cpa-miR160d* is involved in the ripening process of papaya. The miRNA miR396 regulates plant growth and development by inhibiting the expression of growth-regulating factor (GRF) [41], participates in the development of wheat plants [42], and responds to environmental stressors such as cold injury, high temperature, and drought [43,44,45]. In our study, during normal fruit ripening (1 DAT vs. 0 DAT in the control), the expression of *cpa-miR396* was found to be upregulated, while at the early development stage of 1-MCP-delayed ripening (1-MCP 2 h 1 DAT vs. control 1 DAT) in papaya, the expression of *cpa-miR396* was downregulated. In Luan’s (2018) study, by overexpressing *miR172a/b* in tomato plants, the expression of *AP2/ERF* transcription factor was inhibited, and the chlorophyll content and photosynthetic rate increased, as well as the development of higher resistance to phytophthora infection [46]. In the present work, the expression of *cpa-miR172a* showed no significant changes during normal ripening (1 DAT vs. 0 DAT in the control), while during the delayed papaya ripening process (1-MCP 2 h 6 DAT vs. 1 DAT) and the early development stage with abnormal ripening (1-MCP 16 h 1 DAT to control 1 DAT), the expression of *cpa-miR172a* was found to be upregulated. These results indicate that the identified miRNAs may play key roles in papaya fruit ripening.

### 4.3. Degradation Analysis Showed That miRNAs Are Involved in Regulation of Ethylene and Auxin Signaling Pathways

Through miRNA-targeted degradome analysis, miRNAs related to ethylene and auxin signaling were identified, as shown in Figure 6A,B. Their corresponding key target genes are included in Figure 6B: *CpCTR1*, *CpARFs*, *CpTIR1*, and *CpSAUR67*. The miRNAs and their corresponding target genes identified by degradome analysis are shown in Table S8. These miRNAs were found to regulate hormone signal transduction pathways and hormone homeostasis related to developmental processes. CTR1 is located downstream of the ethylene receptor and mediates the signal from the ethylene receptor by negatively regulating the ethylene response. Both *miR1917* and *miR171b* target *CTR1* and are important regulators for ethylene signal transduction in the ripening of tomato fruit [47,48]. In the present study, *CTR1* was targeted by *unconservative_supercontig_75_21810*, *unconservative_supercontig_2_1866*, *unconservative_supercontig_19_9148*, and *unconservative_supercontig_3_2646*. The expression patterns of *unconservative_supercontig_75_21810*, *unconservative_supercontig_19_9148*, and *unconservative_supercontig_3_2646* decreased during fruit ripening. The expression of *unconservative_supercontig_2_1866* decreased in 1-MCP-delayed fruit ripening.

At the early stages of auxin signal transduction, it was found that several gene families, including Aux/IAA (auxin/indole-3-acetic acid), SAUR (small auxin up RNA), and GH3 (Gretchen Hagen3), could respond to auxin treatments. The Aux/IAA protein acts as an important component of the auxin signaling pathway by participating in the regulation of expression for a large number of genes downstream of the auxin signaling pathway by releasing auxin response factor (ARF). Most of the Aux/IAA proteins contain four conserved domains, namely the I, II, III, and IV domains. Domain II is a key component that leads to instability of the Aux/IAA protein, which is later degraded by the ubiquitin-proteasome protein (TIR1) pathway [49,50]. Therefore, TIR1, SAUR, and ARF are key factors in the auxin signaling pathway. In *Arabidopsis thaliana*, *miR393* participates in auxin signal transduction and plant development by regulating *TIR1* [50]. The *miR165/166* miRNA determines the fate of *A*. *thaliana* root cells, is involved in plant hormone cross-talk, and regulates root growth via negative regulation of its target genes, such as the auxin response factors *ARF10*, *ARF16*, and *ARF17* [51]. In the present study, *ARFs* were targeted by *miR156a*, *miR160a*, *miR160d*, *miR319*, and *unconservative_supercontig_182_32956*. The expression of these five miRNAs decreased during fruit ripening. *TIR1* and *SAUR67* were targeted by *miR393* and *unconservative_supercontig_65_20146*, respectively. The expression of *miR393* decreased while that of *unconservative_supercontig_65_20146* increased during papaya fruit ripening.

### 4.4. Network Regulation Diagram of miRNAs and Target Gene Verification

According to the regulatory network diagram, RT-qPCR verification of key miRNAs and target genes was performed, showing that the expression of *cpa-miR172a*, *cpa-miR319*, *cpa-miR390a*, and *cpa-miR396* greatly changed. Previous studies found that miR172a-mediated upregulation of *ERF RAP2-7* during water submergence stress in maize roots restricted plant growth during flood-stress conditions [52]. In the present study, *cpa-miR172a* had an effect on ethylene and auxin signaling pathways. Here, *cpa-miR396* acted on the ethylene signaling pathway, and *cpa-miR319* and *cpa-miR390a* acted on the auxin signaling pathway. Similar results also showed that miR390 targeted ARFs and repressed their expression [53]. It has previously been reported that miR390 and ARFs form an auxin-responsive regulatory network to control lateral root growth in *A. thaliana*. The expression of miR390 is confined to the mesenchymal cells of the xylem prior to lateral root initiation. It was found that miR390 stimulates the production of tasiARFs, which repress the expression of their targets ARF3 and ARF4. There was also positive and negative feedback between ARF2/ARF3/ARF4 and miR390 to regulate lateral root growth [54]. Two highly expressed miRNAs were selected from the ethylene and auxin pathways, and their target genes were verified. We found that the expression signal of GFP protein with target gene binding in tobacco was weakened. These results indicate that *CpARF19-like* and *CpERF RAP2-12-like* are potential targets of cpa-miR390a and cpa-miR396, respectively, and they all may be important in the process of papaya fruit ripening.

## 5. Conclusions

Suitable 1-MCP treatment effectively delays the ripening of papaya fruit, and long-term 1-MCP treatment causes papaya ripening disorder. In this study, after different 1-MCP treatments on papaya fruits at different development stages, miRNA, transcriptome, and degradome analyses were performed. A total of 213 miRNAs and 1741 target genes of these miRNAs were identified. Among these, 11 different miRNAs related to ethylene and auxin and 12 corresponding target genes were found. The analysis and verification of *cpa-miR390a* and *cpa-miR396*, targeting *CpARF19-like* and *CpERF RAP2-12-like*, respectively, highlighted that these miRNAs and their target genes are likely partners in the ethylene and auxin signaling pathways. Our results indicate that these miRNAs may play an important role in regulating fruit ripening by targeting ethylene and auxin signaling pathways. Unsuitable 1-MCP treatment may disruptively repress miRNA function and cause fruit ripening disorder.

## Figures and Tables

**Table 1:** Expression profiles of DEMs and the predicted target genes under different conditions.

	miRNA	miR_Regulate	Predicted Target Gene ID	Predicted Target Gene Name
CK 1 d vs. 0 d	cpa-miR319	Down	evm.TU.supercontig_7.3	*Carica papaya* auxin response factor 3
	cpa-miR396	Up	evm.TU.supercontig_48.26	*Carica papaya* 1-aminocyclopropane-1-carboxylate synthase 1-like
	cpa-miR396	Up	evm.TU.supercontig_481.1	*Carica papaya* ethylene-responsive transcription factor RAP2-12-like
	cpa-miR8140	Up	evm.TU.supercontig_1322.1	*Carica papaya* 1-aminocyclopropane-1-carboxylate synthase 1-like
CK 6 d vs. 0 d	cpa-miR160d	Down	evm.TU.supercontig_49.122	*Carica papaya* auxin response factor 17
	cpa-miR160d	Down	evm.TU.supercontig_53.88	*Carica papaya* auxin response factor 16-like
	cpa-miR160d	Down	evm.TU.supercontig_65.4	*Carica papaya* auxin response factor 10
2 h 6 d vs. CK 6 d	cpa-miR390a	Up	evm.TU.supercontig_261.2	*Carica papaya* auxin response factor 19-like
16 h 1 d vs. CK 1 d	cpa-miR172a	Up	C.papaya_newGene_850	*Carica papaya* auxin transport protein BIG
	cpa-miR172a	Up	evm.TU.supercontig_1.271	*Carica papaya* ethylene-responsive transcription factor RAP2-7-like
	cpa-miR172a	Up	evm.TU.supercontig_114.55	*Carica papaya* ethylene-responsive transcription factor RAP2-7-like
16 h 6 d vs. CK 6 d	cpa-miR160d	Up	evm.TU.supercontig_49.122	*Carica papaya* auxin response factor 17
	cpa-miR160d	Up	evm.TU.supercontig_53.88	*Carica papaya* auxin response factor 16-like
	cpa-miR160d	Up	evm.TU.supercontig_65.4	*Carica papaya* auxin response factor 10
	unconservative_supercontig_2_1866	Up	evm.TU.supercontig_7.3	*Carica papaya* auxin response factor 3
2h 6 d vs. 1 d	cpa-miR172a	Up	C.papaya_newGene_850	*Carica papaya* auxin transport protein BIG
	cpa-miR172a	Up	evm.TU.supercontig_1.271	*Carica papaya* ethylene-responsive transcription factor RAP2-7-like
	cpa-miR172a	Up	evm.TU.supercontig_114.55	*Carica papaya* ethylene-responsive transcription factor RAP2-7-like
	cpa-miR396	Down	evm.TU.supercontig_48.26	*Carica papaya* 1-aminocyclopropane-1-carboxylate synthase 1-like
	cpa-miR396	Down	evm.TU.supercontig_481.1	*Carica papaya* ethylene-responsive transcription factor RAP2-12-like
16 h 6 d vs. 1 d	cpa-miR167c	Up	evm.TU.supercontig_17.52	*Carica papaya* auxin response factor 6
	cpa-miR8140	Up	evm.TU.supercontig_1322.1	*Carica papaya* 1-aminocyclopropane-1-carboxylate synthase 1-like

**Table 2:** Expression profiles of DEMs and their target genes under small RNAs and transcriptome sequencing.

	miRNA	miR_Regulate	Target Gene ID	Target Gene Name	Target Gene Regulation
CK 1 d vs. 0 d	cpa-miR396	Up	evm.TU.supercontig_48.26	*Carica papaya* 1-aminocyclopropane-1-carboxylate synthase 1-like	Down
	cpa-miR396	Up	evm.TU.supercontig_481.1	*Carica papaya* ethylene-responsive transcription factor RAP2-12-like	Down
	cpa-miR8140	Up	evm.TU.supercontig_1322.1	*Carica papaya* 1-aminocyclopropane-1-carboxylate synthase 1-like	Down
	unconservative_supercontig_120_28048	Up	evm.TU.contig_32826	*Carica papaya* indole-3-acetic acid-amido synthetase GH3.6	Down
	unconservative_supercontig_46_16464	Up	evm.TU.supercontig_2.209	*Carica papaya* 1-aminocyclopropane-1-carboxylate oxidase homolog 4-like	Down
	cpa-miR319	Down	evm.TU.supercontig_7.3	*Carica papaya* auxin response factor 3	Up
CK 6 d vs. 0 d	unconservative_supercontig_46_16464	Up	evm.TU.supercontig_2.209	*Carica papaya* 1-aminocyclopropane-1-carboxylate oxidase homolog 4-like	Down
	cpa-miR160d	Down	evm.TU.supercontig_49.122	*Carica papaya* auxin response factor 17	Up
	cpa-miR160d	Down	evm.TU.supercontig_53.88	*Carica papaya* auxin response factor 16-like	Up
	cpa-miR160d	Down	evm.TU.supercontig_65.4	*Carica papaya* auxin response factor 10	Up
	unconservative_supercontig_2_1866	Down	evm.TU.supercontig_7.3	*Carica papaya* auxin response factor 3	Up
2h 6 d vs. CK 6 d	cpa-miR390a	Up	evm.TU.supercontig_261.2	*Carica papaya* auxin response factor 19-like	Down
16 h 1 d vs. CK 1 d	cpa-miR172a	Up	C.papaya_newGene_850	*Carica papaya* auxin transport protein BIG	Down
	cpa-miR172a	Up	evm.TU.supercontig_1.271	*Carica papaya* ethylene-responsive transcription factor RAP2-7-like	Down
	cpa-miR172a	Up	evm.TU.supercontig_114.55	*Carica papaya* ethylene-responsive transcription factor RAP2-7-like	Down
	cpa-miR172a	Up	evm.TU.supercontig_139.43	*Carica papaya* ethylene-responsive transcription factor RAP2-7	Down
	unconservative_supercontig_120_28048	Down	evm.TU.contig_32826	*Carica papaya* indole-3-acetic acid-amido synthetase GH3.6	Up
	unconservative_supercontig_52_17983	Up	evm.TU.supercontig_83.80	*Carica papaya* ethylene-responsive transcription factor 4	Down
16 h 6 d vs. CK 6 d	cpa-miR160d	Up	evm.TU.supercontig_49.122	*Carica papaya* auxin response factor 17	Down
	cpa-miR160d	Up	evm.TU.supercontig_53.88	*Carica papaya* auxin response factor 16-like	Down
	cpa-miR160d	Up	evm.TU.supercontig_65.4	*Carica papaya* auxin response factor 10	Down
	unconservative_supercontig_2_1866	Up	evm.TU.supercontig_7.3	*Carica papaya* auxin response factor 3	Down
2 h 6 d vs. 1 d	cpa-miR172a	Up	C.papaya_newGene_850	*Carica papaya* auxin transport protein BIG	Down
	cpa-miR172a	Up	evm.TU.supercontig_1.271	*Carica papaya* ethylene-responsive transcription factor RAP2-7-like	Down
	cpa-miR172a	Up	evm.TU.supercontig_114.55	*Carica papaya* ethylene-responsive transcription factor RAP2-7-like	Down
	cpa-miR172a	Up	evm.TU.supercontig_139.43	*Carica papaya* ethylene-responsive transcription factor RAP2-7	Down
	cpa-miR396	Down	evm.TU.supercontig_48.26	*Carica papaya* 1-aminocyclopropane-1-carboxylate synthase 1-like	Up
	cpa-miR396	Down	evm.TU.supercontig_481.1	*Carica papaya* ethylene-responsive transcription factor RAP2-12-like	Up
	unconservative_supercontig_9_5033	Down	evm.TU.supercontig_9.126	*Carica papaya* auxin-binding protein T85	Up
16 h 6 d vs. 1 d	cpa-miR167c	Up	evm.TU.supercontig_17.52	*Carica papaya* auxin response factor 6	Down
	cpa-miR8140	Up	evm.TU.supercontig_1322.1	*Carica papaya* 1-aminocyclopropane-1-carboxylate synthase 1-like	Down

## Data Availability

The data presented in this study are available on request from the corresponding author.

## Supplementary Materials

The following are available online at https://www.mdpi.com/article/10.3390/foods10071643/s1, Figure S1: Histogram of COG classification; Table S1: RT-qPCR primers of miRNAs; Table S2: RT-qPCR primers of target genes; Table S3: Primers for plant expression vectors; Table S4: Sequencing data statistics table; Table S5: miRNA statistical results of each sample; Table S6: miRNA expression level statistics table; Table S7: miRNAs and their targets in degradome analysis; Table S8: miRNAs and their corresponding target genes of the degradome analysis.
