# Supplementary figures and images for: Small RNAs, Degradome, and Transcriptome Sequencing Provide Insights into Papaya Fruit Ripening Regulated by 1-MCP
Source: Foods. 2021 Jul 15;10(7):1643. doi: 10.3390/foods10071643 (PMC8303378; doi:10.3390/foods10071643)

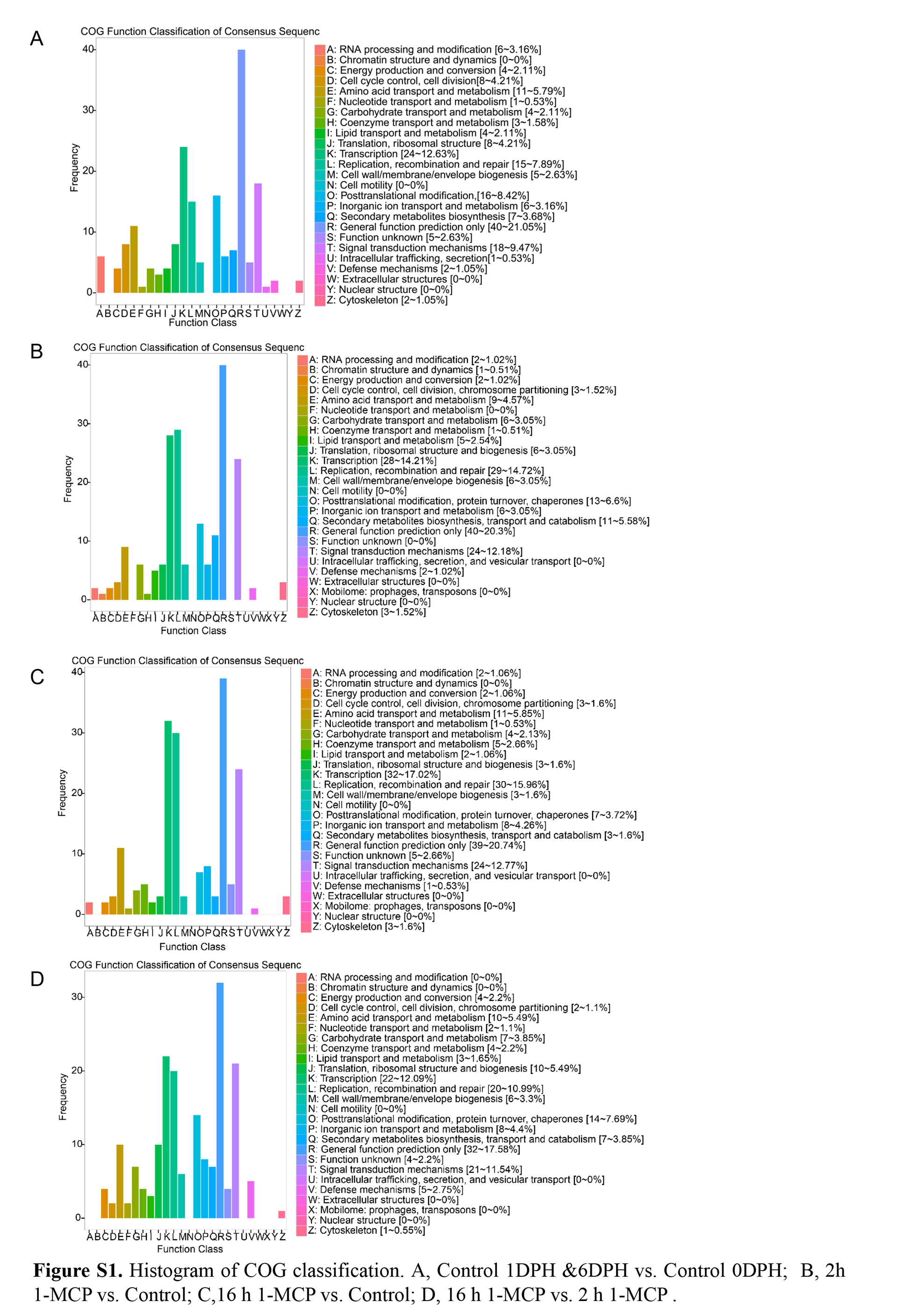

Supplement: Supplementary file 1 [file foods-10-01643-s001.zip › Figure S1. Histogram of COG classification.tif]
